# Probiotics, Prebiotics, and Phytogenic Substances for Optimizing Gut Health in Poultry

**DOI:** 10.3390/microorganisms10020395

**Published:** 2022-02-08

**Authors:** Awad A. Shehata, Sakine Yalçın, Juan D. Latorre, Shereen Basiouni, Youssef A. Attia, Amr Abd El-Wahab, Christian Visscher, Hesham R. El-Seedi, Claudia Huber, Hafez M. Hafez, Wolfgang Eisenreich, Guillermo Tellez-Isaias

**Affiliations:** 1 Research and Development Section, PerNaturam GmbH, 56290 Gödenroth, Germany; 2 Avian and Rabbit Diseases Department, Faculty of Veterinary Medicine, University of Sadat City, Sadat City 32897, Egypt; 3 Department of Animal Nutrition and Nutritional Diseases, Faculty of Veterinary Medicine, Ankara University (AU), 06110 Ankara, Turkey; sayalcin@ankara.edu.tr; 4 Department of Poultry Science, University of Arkansas, Fayetteville, AR 72701, USA; jl115@uark.edu; 5 Clinical Pathology Department, Faculty of Veterinary Medicine, Benha University, Benha 13518, Egypt; shereenbh@yahoo.com; 6 Department of Agriculture, Faculty of Environmental Sciences, King Abdulaziz University, Jeddah 21589, Saudi Arabia; yaattia@kau.edu.sa; 7 Institute for Animal Nutrition, University of Veterinary Medicine Hannover, 30173 Hannover, Germany; amrwahab5@mans.edu.eg (A.A.E.-W.); christian.visscher@tiho-hannover.de (C.V.); 8 Department of Nutrition and Nutritional Deficiency Diseases, Faculty of Veterinary Medicine, Mansoura University, Mansoura 35516, Egypt; 9 Pharmacognosy Group, Biomedical Centre, Department of Pharmaceutical Biosciences, Uppsala University, SE 75124 Uppsala, Sweden; hesham.el-seedi@farmbio.uu.se; 10 International Research Center for Food Nutrition and Safety, Jiangsu University, Zhenjiang 212013, China; 11 International Joint Research Laboratory of Intelligent Agriculture and Agri-Products Processing, Jiangsu Education Department, Jiangsu University, Zhenjiang 212013, China; 12 Bavarian NMR Center, Structural Membrane Biochemistry, Department of Chemistry, Technische Universität München, Lichtenbegstr. 4, 85748 Garching, Germany; claudia.huber@tum.de (C.H.); wolfgang.eisenreich@mytum.de (W.E.); 13 Institute of Poultry Diseases, Faculty of Veterinary Medicine, Free University of Berlin, 14163 Berlin, Germany; hafez.mohamed@fu-berlin.de

**Keywords:** gut microbiota, dysbiosis, tight junctions, synbiotics, phytogenic substances, nutraceuticals, poultry, feed additives

## Abstract

The gut microbiota has been designated as a hidden metabolic ‘organ’ because of its enormous impact on host metabolism, physiology, nutrition, and immune function. The connection between the intestinal microbiota and their respective host animals is dynamic and, in general, mutually beneficial. This complicated interaction is seen as a determinant of health and disease; thus, intestinal dysbiosis is linked with several metabolic diseases. Therefore, tractable strategies targeting the regulation of intestinal microbiota can control several diseases that are closely related to inflammatory and metabolic disorders. As a result, animal health and performance are improved. One of these strategies is related to dietary supplementation with prebiotics, probiotics, and phytogenic substances. These supplements exert their effects indirectly through manipulation of gut microbiota quality and improvement in intestinal epithelial barrier. Several phytogenic substances, such as berberine, resveratrol, curcumin, carvacrol, thymol, isoflavones and hydrolyzed fibers, have been identified as potential supplements that may also act as welcome means to reduce the usage of antibiotics in feedstock, including poultry farming, through manipulation of the gut microbiome. In addition, these compounds may improve the integrity of tight junctions by controlling tight junction-related proteins and inflammatory signaling pathways in the host animals. In this review, we discuss the role of probiotics, prebiotics, and phytogenic substances in optimizing gut function in poultry.

## 1. Introduction

The permeability of the intestinal tract controls the uptake of nutrients and the transport of unwanted extracellular substances such as bacteria and xenobiotics, in addition to the non-digested substances. Therefore, gut health plays an essential role in the pathogenesis of various intestinal disorders. The permeability of the intestine is controlled by gut microbiota, digestive secretions, physical barriers (mucin, intestinal epithelial cells lining and tight junctions), and chemicals such as cytokines [1].

Under normal conditions, the symbiotic relationship between the gut microbiota and the host crucially determines intestinal health. However, a disturbance in the gut microbiota can lead to an imbalanced host–microbe relationship, which is called “dysbiosis” [2]. Several factors, such as antinutritional factors in feed, heavy metals, toxic substances, bacterial toxins, herbicides, and antibiotics, can disrupt the gut microbiota. These impacts can lead to localized inflammation, extensive infection, or even intoxication [3,4,5], Additionally, the intestinal epithelium forms tight connections, acting as a biological barrier that controls the paracellular transit of different materials across the intestinal epithelium, including ions, solutes, and water. It also functions as a barrier of extracellular bacteria, antigens, and xenobiotics.

The impaired intestinal barrier function, commonly known as “leaky gut”, is a condition in which the small intestine lining becomes damaged, leading to infiltration of luminal contents such as bacteria and their associated components including toxins to pass between epithelial cells. These conditions subsequently lead to cell damage and/or inflammation of the intestine, characterized by increased levels of bacteria-derived endotoxins in blood. This inflammatory process consumes significant amounts of nutrients, and, subsequently, has negative effects on metabolic responses, in particular on immunometabolic and endocrine responses. As a result, animal performances are severely reduced [6].

Additionally, field observations in Europe showed that the poultry industry faced several problems after the ban of antibiotic growth promoters (AGPs), including negative impacts on performance, animal welfare aspects, and general health issues [7]. In response to the AGP ban, several alternatives to antibiotics, such as probiotics, prebiotics, and phytogenic substances, have been developed, tested, evaluated, and used for chicken and turkey production at an increasing frequency [8]. In this review, we discuss the role of these alternatives in maintaining gut function through modulation of the gut microbiota and the related effects benefitting health and quality of poultry.

## 2. Intestinal Microbiota in Poultry

Microorganisms that live in animals’ gastrointestinal tracts (GITs) are a prime example of beneficial bacteria [9]. Indeed, the GIT is the home of a diverse and plentiful microbial community providing essential functions to their host animals. Although the intestine is exposed to microflora components from birth or hatching, little is known about their impact on healthy development and function. Microorganisms are more densely populated in the GIT than in any other organ [9]. Animals have evolved the ability to host complex and dynamic consortia of microbes over their life cycle during millions of years of evolution [10].

**Figure 1:**
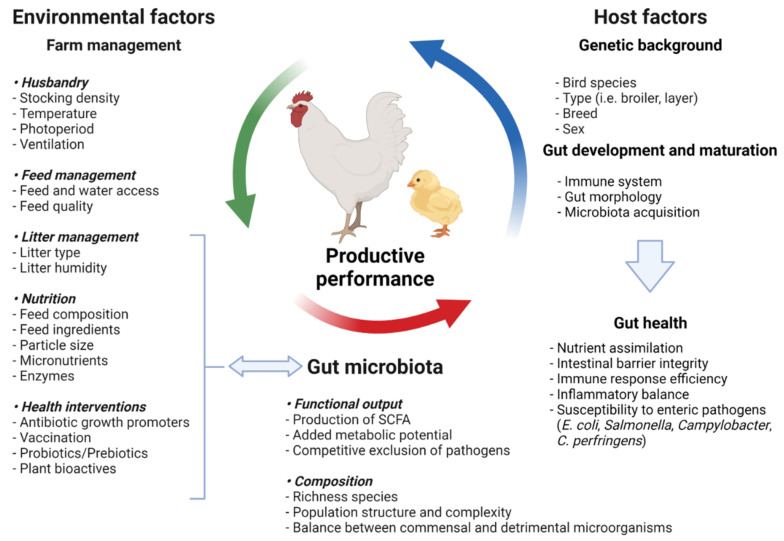
Factors affecting the gut microbiota composition modified according to Carrasco et al. [21] (figure was created with BioRender.com, accessed on 15 December 2021).

As a result, a detailed understanding of the contributions of these indigenous microbial communities to host development and adult physiology is required for a thorough comprehension of vertebrate biology [11]. Animal species, breed, age, nutrition, environment, rearing forms, stocking density, stress, and medicine can all have an impact on the delicate composition of the gut microbiota [12]. Factors affecting the composition in gut microbiota are shown in Figure 1. Most of these intestinal microflora’s species cannot be cultured when they are removed from their niches, as is the case with most complex ecosystems.

Colonization of avian guts could already start during embryogenesis [13] and progresses to the formation of a complex and dynamic microbial society [14]. Based on principles established during animal history, extensive and combinatorial microbial–microbial and host–microbial interactions are likely to govern the microbiota assembly [15]. Comparing germ-free rodents that were raised without exposure to microorganisms to those that built up a microbiota since birth, or those that were colonized with microbiota components during or after postnatal development, a variety of host functions influenced by indigenous microbial communities were identified [16].

The microbiota, for example, directs the formation of gut-associated lymphoid tissue, aids immune system education, affects the integrity of the intestinal mucosal barrier, modulates proliferation and differentiation of epithelial lineages, regulates angiogenesis, modifies enteric nervous system activity, and plays a critical role in extracting and processing the nutrients consumed [17,18]. Proteins and protein breakdown products, sulfur-containing substances, and endogenous or foreign glycoproteins can all be metabolized by the microflora [19]. Some bacteria even feed on bacterial fermentation products or intermediates including H_2_, lactate, succinate, formate, and ethanol and convert them to end products which are again secreted to the gut lumen, such as short-chain fatty acids (SCFA), a process that has a direct impact on gut physiology [20].

More than 90% of all gut microbiota species in humans and animals belong to the phyla *Bacteroidetes*, *Firmicutes* and *Actinobacteria*, others are *Fusobacteria*, *Proteobacteria*, *Verrucomicrobia*, and *Cyanobacteria* [22,23]. In chickens, the phyla *Bacteroidetes* and *Firmicutes* are the most predominant representatives in the gut. In human and several animals, the ratio between *Firmicutes* and *Bacteroidetes* is a health/metabolism-associated marker [24,25,26,27]. *Firmicutes* species decompose polysaccharides and produce butyrate, and *Bacteroidetes* species degrade complex carbohydrates and synthesize mainly propionate [25]. The mechanisms by which bacteria exert effects on the gastrointestinal tract are largely unknown, but manipulation of these triggers is considered to be a promising mean to achieve optimal health and performance [28,29]. It is also assumed that the molecular principles that aid in the modification and maintenance of normal physiological functioning of the gut microbiota are mainly derived from food and its supplements, such as nutraceuticals [30]. Nutraceuticals can include everything from isolated nutrients (vitamins, minerals, amino acids, fatty acids) to herbal goods (polyphenols, herbs, spices), dietary supplements (probiotics, prebiotics, synbiotics, organic acids, antioxidants, enzymes), and genetically modified foods. These nutraceuticals also aid in the prevention of infectious diseases of the host [31]. Additionally, several multidrug resistance bacteria have emerged making, this crisis global [32,33,34]. Nutraceuticals will be required to reduce the use of antibiotics [35].

Lactic acid bacteria have been used as feed supplements since pre-Christian times when humans ingested fermented milk. This subject was not analyzed scientifically until the last century, when Eli Metchnikoff (1845–1916), working at the Pasteur Institute in Paris, discovered a link between human longevity and the importance of maintaining a healthy mix of beneficial and pathogenic microbes in the gut. Elie Metchnikoff received the Nobel Prize in Physiology in 1908 for discovering the role of phagocytes and other components in the immune system, but his correct description of key constituents in the body’s gut flora is also noteworthy [36]. He devised and administered bacteriotherapy, or the use of lactic acid bacteria in food regimens, to his patients. He also highlighted the fact that Bulgarian peasants consumed a lot of spoiled milk and lived long lives [36]. From spoiled milk, Metchnikoff and his co-workers identified the ‘Bulgarian bacillus,’ most likely *Lactobacillus bulgaricus*, which was employed in later trials.

Today, this microorganism is known as *Lactobacillus delbrueckii* subsp. *bulgaricus*, which is one of the bacteria that is used to ferment milk and make yogurt. Following Metchnikoff’s death in 1916, the focus of work in this field shifted to the United States. In the late 1940s it was discovered that antibiotics added to farm animals’ feed aided their growth [37]. The need to understand the mechanisms behind this impact prompted more research into the composition of the gut microflora and how it can affect the host animal health.

**Figure 2:**
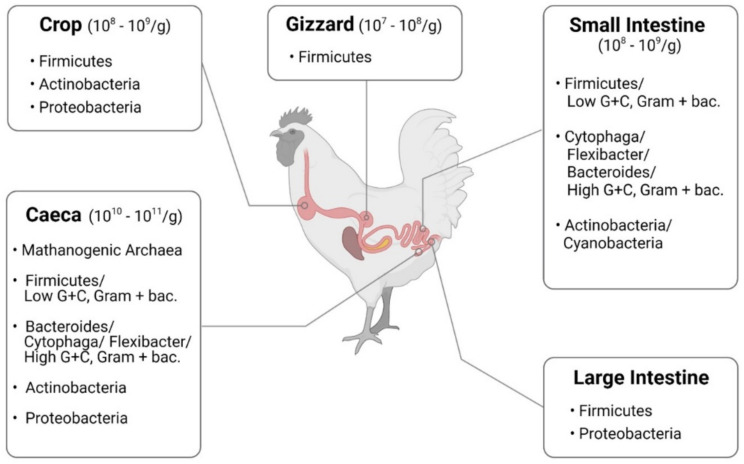
Microbiota in chickens, summarized from Shang et al. [40] (figure was created with BioRender.com, accessed on 15 December 2021).

Progress in bacteriology and the easier availability of germ-free animals helped to assess the impact of newly identified intestinal occupants on the host [38]. Based on these studies, it became clear that *Lactobacillus acidophilus* was not the only *Lactobacillus* in the intestine, and a variety of other species were examined and eventually included in probiotic formulations. The main representatives in gut microbiota of chickens are summarized in Figure 2. Understanding how the intestine matures and develops in chickens and how feed supplements benefit the gut performance will increase feed efficiency, growth, and overall health [39].

## 3. Intestinal Barrier and Tight Junctions

**Figure 3:**
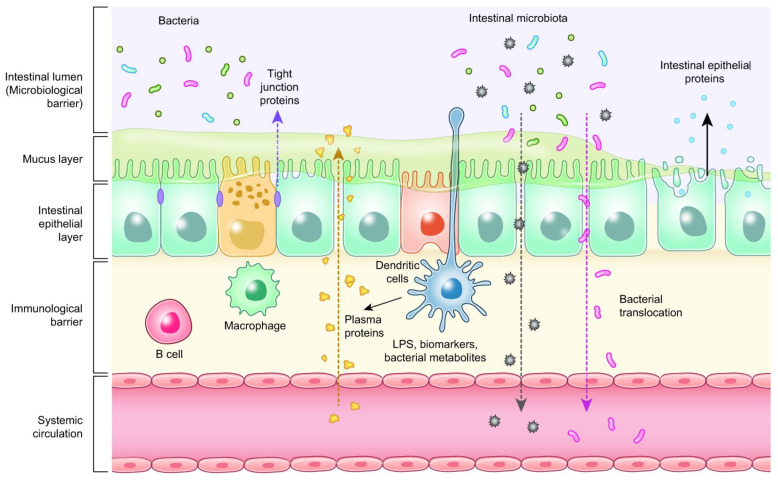
Intestinal epithelial barrier and intestinal microbiota interaction.

Enterocytes are the cornerstone of the intestinal mucosal monolayer that protects the host from the external environment. A scheme of the intestinal epithelial barrier and some interactions with intestinal microbiota is shown in Figure 3. Enterocytes are connected by the so-called tight junctions (TJs), which constitute a continuous belt of intimate contacts formed during the assembly process of integral transmembranes (occludin, claudins, junctional adhesion molecules (JAMs), and tricellulin) and peripheral membranes (zonula occludens-1 (ZO-1), ZO-2, and ZO-3). The TJ proteins are located between adjacent enterocytes, sealing the paracellular space and regulating the permeability of the intestinal barrier. Therefore, these proteins prevent the transit of microorganisms, toxins and other antigens from the intestinal lumen to the systemic circulation [41,42]. The formation and function of tight junctions are controlled by intracellular signal transduction pathways: (i) protein kinase C (PKC), A (PKA), and G (PKG) signaling, (ii) phosphatase-Rho, myosin light chain (MLC) kinase (MLCK), MAPK signaling, and (iii) the PI3K/Akt pathway [43,44].

The disruption of tight junctions by bacterial factors can occur in the following steps: (i) bacterial lipopolysaccharide (LPS) activates the intestinal epithelial cells and macrophages; (ii) these cells secrete proinflammatory cytokines such as IL-1ß; and (iii) IL-1ß further activates these cells and triggers intracellular signaling, such as p38 MAP kinase, which subsequently activates MLCK. Finally, these processes lead to an increase in intestinal permeability [45,46]. Thus, leaky gut syndrome develops as a response to pathogens, feed deprivation, and stress [47,48,49,50].

## 4. Biomarkers Related to Intestinal Health of Animals

The interactions between the epithelial barrier function, intestinal inflammation, and the microbial environment influence gut health [51,52]. Therefore, the discovery of reliable, widespread biomarkers to measure intestinal inflammation and barrier function is an important ongoing area of research. A summary of some of the known biomarkers related to intestinal health is presented in Table 1. To study intestinal health, it is also important to develop inflammatory gut models with different challenge conditions (anti-nutritional factors, pathogens, toxins, and environmental triggers) [53,54]. Inflammation can also be associated with oxidative stress and changes in the expression of genes related to oxidative stress, indicating that oxidative stress may have a critical role in the physiological intestinal function [55]. One quantitative technique that is used to evaluate the integrity of tight junction proteins in epithelial cell monolayers is the measurement of transepithelial electrical resistance (TEER) [56]. Mitochondrial respiration is required to maintain TEER, implying that oxidation plays a critical role in Caco-2 cell tight junction stability [57]. According to Janssen-Duijghuijsen et al. [57], reduced mitochondrial ATP production resulted in a decrease of intestinal permeability and an increase in occludin and claudin-1 gene expression, but a decrease in claudin-2 and claudin-7 gene expression. Consequently, a direct connection between mitochondrial function, cellular energy status, and intestinal integrity was established. Often, oxidative stress is quantified by examining metabolites formed during or after an oxidative process. An antioxidant enzyme that detoxifies harmful metabolic by-products and that is usually measured as a biomarker is superoxide dismutase (SOD) [58]. Other biomarkers that could be used to measure antioxidant activity include thiobarbituric acid reactive substances (TBARS), which are metabolites formed during peroxidation; total antioxidant capacity; and the Griess assay, which utilizes nitrite and nitrate breakdown to determine the concentration of nitric oxide within the cell

Biomarkers for the evaluation of intestinal health can also be related to monitoring intestinal function. Citrulline is a nitrogen-containing by-product of glutamine metabolism that can be converted to arginine and is produced mainly by enterocytes of the small intestine [60]. Plasma citrulline levels have been associated with intestinal absorption of markers such as mannitol in pre-weaned piglets, indicating that citrulline may be utilized to monitor intestinal function [61]. The extracellular signal-regulated kinase (ERK) is another biomarker that can be considered to be an option because it serves as a critical signaling pathway for intestinal epithelial proliferation and tissue healing. Thus, serum ERK activity can reflect intestinal disruption caused by a stressor [62].

In the case of biomarkers related to the immune activity that can influence intestinal health, secretory IgA (SIgA) is a critical component of the humoral immune system and the leading immunoglobulin that interacts with pathogens on the mucosa surface. Consequently, it has a close relationship with the homeostasis of the intestinal environment [63]. A proinflammatory cytokine with immunostimulatory and immunomodulatory properties is interferon-gamma (INF-γ). This cytokine has been related to the endocytosis of tight junction proteins. Hence, it has a feasible impact on intestinal permeability [64,65]. Ultimately, both innate and adaptive immune responses are likely to provide viable biomarkers for assessing intestinal health.

The histomorphological analysis is another type of evaluation closely influenced by an adequate balance of the intestinal environment. Villus height, crypt depth, and the villus height to crypt depth ratio are parameters that can be used to calculate the area of absorption in the different sections of the intestine, and at the same time be indicative of the epithelial cell turnover in the intestinal barrier [66].

Bacterial translocation and gene expression of TJ such as claudins, occludins, and zonula occludens (ZO-1) are intestinal permeability biomarkers used to evaluate gut health. Bacterial translocation has been related to diseases such as chondronecrosis with osteomyelitis in broiler and broiler breeders [67,68], suggesting the migration of enteric pathogens to the thoracic vertebrae. TJs such as occludin have shown to be downregulated in human patients with inflammatory bowel diseases (Crohn’s disease), and in chickens under nutritional gut health challenge condition models [69,70], therefore revealing the fundamental role of TJs such as occludin in maintaining intestinal barrier integrity. Another well-known biomarker that has been utilized in poultry to evaluate intestinal permeability is the measurement of fluorescein isothiocyanate dextran (FITC-d) in the serum. During intestinal inflammation, the disruption of the TJ proteins allows the FITC-d molecule to diffuse into systemic circulation, allowing measurement of this biomarker under different challenging conditions, including 24 h of fasting in broiler chickens [71].

A different set of biomarkers candidates include the fatty acid binding proteins (FABP), which are intracellular lipid chaperones in charge of orchestrating lipid metabolism and critical lipid-sensitive pathways in macrophages and adipocytes [72]. FABP2 has been studied in humans [73] and in chickens [54], showing a downregulation response when there is intestinal barrier injury. Some non-invasive biomarkers that are currently being studied in fecal samples by different research groups are fibronectin, calprotectin, and lipocalin [74]. These biomarker candidates have shown promising results in chickens; nevertheless, there have been also inconsistencies between studies. Ultimately, the objective is to continue searching for intestinal health biomarkers that can be easily measured from samples that do not require an extensive preparation time or cost.

## 5. Probiotics

Properly dosed probiotics improve gut microbial balance, colonization resistance against infections, and immunological responses [75]. *Lactobacillus* spp., *Streptococcus thermophilus*, *Enterococcus faecalis*, and *Bifidobacterium* spp. are the most frequent lactic acid bacteria (LAB) utilized in probiotic formulations. Possible mechanisms of action include: (i) maintaining a healthy balance of bacteria in the gut by competitive exclusion, i.e., in a process by which beneficial bacteria exclude potential pathogenic bacteria via competition for attachment sites in the intestine and nutrients, and (ii) preventing bacterial overgrowth in the gut [76].

There is also ample evidence that probiotics affect the immune system by balancing pro- and anti-inflammatory cytokines [77]. Some probiotics have antioxidant capabilities and improve barrier integrity [78]. Another study found that both innate and humoral immunity are improved while using probiotics [79]. A commercial lactic acid bacteria-based probiotic (FloraMax PW Boehringer Ingelheim) for poultry use was studied recently. Using this specified LAB culture, extensive laboratory and field research has shown greater resistance to *Salmonella* sp. infections in hens and turkeys [75,76,77,78]. Several probiotic strains improved the animal performance and could be used as potential alternatives to antibiotics [80,81,82,83,84,85,86]. Higgins et al., reported that probiotics reduce idiopathic diarrhea in commercial turkey brooding houses, according to published experimental and commercial trials [83]. Additionally, probiotic blend was shown to improve performance and reduce production costs in large-scale commercial experiments [81,84]. Probiotic-treated birds demonstrated variations in gene expression related to the nuclear factor kappa B (NF-κB) complex, according to recent microarray research [85]. These findings indicate that specified probiotic cultures may occasionally be an attractive alternative to traditional antibiotic therapy [86].

Commercial probiotics that are shelf-stable, cost-effective, and feed-stable (resistance to the heat pelletization process) are urgently needed to promote compliance and wider usage. Some probiotic products contain bacterial spore formers, typically of the *Bacillus* genus. Some (but not all) have been proven to prevent certain gastrointestinal problems. The variety of species employed and their multiple uses are astounding. These means prove the benefit that some *Bacillus* spore isolates are the most heat-tolerant spores known and can thus also be employed in intense heat circumstances [87]. Thus, spores from selected *Bacillus* strains have been used as a reliable direct feed microorganisms (DFM) in animal production due to their ability to withstand harsh environmental conditions and long storage periods [88].

Field trials suggested that one strain of *Bacillus subtilis* spore isolate is as effective as FloraMax PW in reducing *Salmonella* spp. [89,90]. Further research may reveal further potent isolates or combinations of isolates. Some of these environmental *Bacillus* isolates have been tested in vitro for antibiotic activity, heat stability, and population growth. Improving amplification and sporulation efficiency is critical to gain industrial approval of a feed-based probiotic for ante-mortem food-borne pathogen intervention. An enhanced vegetative growth and sporulation rate may lead to new efficiencies for commercial amplification and cost-effective product creation at very high spore counts [91].

*Bacillus*-DFM has also been shown to prevent GIT disorders and provide a variety of nutritional benefits to both animals and humans [92]. In vitro and in vivo studies have shown that 90% of *B. subtilis* spores germinate in different segments of the GIT within 60 min in the presence of feed [93]. Moreover, using different poultry diets in vitro (rye, wheat, barley, and oat based-diets), the inclusion of selected *Bacillus*-DFM candidates that produce a different set of extracellular enzymes resulted in a significant reduction in both digesta viscosity and *Clostridium perfringens* proliferation between control and *Bacillus*-DFM supplemented diets [70].

The increased digesta viscosity and longer feed passage time caused by high soluble non-starch polysaccharide (NSP) concentrations in poultry diets influence the intestinal bacterial population [94]. Hence, exogenous carbohydrases (xylanase, glucanase, mannanase, galactosidase, and pectinase) are used as feed additives in an attempt to reduce the negative impact of these anti-nutritional factors [94,95]. Interestingly, it was shown that the supplementation of the *Bacillus*-based DFM improved growth performance, digesta viscosity, bacterial translocation, microbiota composition, and bone mineralization in broiler chickens and turkey fed a rye-based diet [70,96]. These differences may be due to fewer substrates available for bacterial growth, resulting in less intestinal inflammation and bacterial translocation when intestinal viscosity was reduced by including the DFM candidate, implying that supplemented groups absorbed more nutrients through the intestinal brush border. The significant improvements in performance observed in turkeys and chickens fed the *Bacillus*-DFM supplemented diet when compared to the unsupplemented control group suggests that the production of enzymes from the combined *Bacillus* spp. strains used as DFM can increase nutrient absorption, promoting growth performance and a more efficient feed conversion ratio, in addition to improving intestinal integrity [70,96]. It was shown that this DFM significantly reduces the severity of *Salmonella enterica* serovar Enteritidis experimental infections [97] and aflatoxicosis [98].

Bacterial translocation, intestinal viscosity, microbiota composition, and bone mineralization in broiler chickens were shown to be affected by rye energy use in one study [44]. However, the *Bacillus*-DFM reverses the negative effects of high NSP diets in poultry [63,94]. Additionally, the performance of broiler chicks and turkey poults was improved by DFM inclusion in reduced fat diets, which was associated with increased energy digestibility as measured by apparent metabolizable energy and nitrogen corrected [99].

## 6. Prebiotics

Prebiotics are a relatively recent concept, arising from the idea that nondigestible food elements (e.g., nondigestible oligosaccharides) are selectively fermented by bacteria known to benefit gut function [75]. The proliferation of endogenous lactic acid bacteria and *Bifidobacteria* in the gut has been demonstrated to benefit host health [39]. Prebiotics may help *Bifidobacteria* and *lactobacilli* to proliferate in the gut, enhancing the host microbial balance. Prebiotics, unlike probiotics, encourage the gut bacteria that have acclimated to the gastrointestinal tract’s environment [100]. Other gastrointestinal alterations include increased intestine length in elderly humans [101]. Prebiotics alter the colonic microbiota and may impact gut metabolism in humans [102]. Healthy gut microbiota may increase absorption, protein metabolism, energy metabolism, fiber digestion, and gut maturation in Leptin-Resistant Mice [103]. Prebiotics have also been shown to improve host defense and reduce pathogen-induced mortality in birds [104].

Prebiotics’ ability to increase the quantity of LAB in the gut may aid in the competitive exclusion of pathogens from the gastrointestinal tract of birds [39]. The increased intestinal acidity caused by prebiotics may also help to reduce infections in the gut of chickens. Prebiotics have also been shown to boost the immunological response in chickens, resulting in faster infection clearance [105]. For example, prebiotics may directly interact with gut immune cells or indirectly interact with immune cells via preferred colonization of beneficial bacteria and microbial metabolites [106,107]. Prebiotics may work similarly to probiotics in supporting chicken intestinal health [73]. Inulin, fructo-oligosaccharides (FOS), mannan-oligosaccharides (MOS), galacto-oligosaccharides (GOS), soya-oligosaccharides (SOS), xylo-oligosaccharides (XOS), pyrodextrins, isomalto-oligosaccharides (IMO), and lactulose are the most commonly utilized prebiotics in poultry [103].

Prebiotic research on poultry has been conducted since 1990, resulting in a large library of studies. Prebiotics in broiler feed have been demonstrated to boost lactobacilli levels. Some investigations on the microbial effects of prebiotic supplementation found increased *Bifidobacteria* and decreased clostridia [108]. *Salmonella* and coliforms were reduced by some [109,110]. The use of prebiotics has also been shown to reduce harmful bacteria such as streptococci and staphylococci in infants [111]. Prebiotics increased intestinal villus height in broiler diets, according to intestinal morphology. Detoxification and elimination processes are enhanced by a healthy population of these helpful bacteria in the digestive tract [112]. Prebiotics have been demonstrated to improve eggshell and bone quality, improve mineral utilization, and improve performance in egg-laying hens [113].

A frequently used prebiotics concerns *Aspergillus oryze*, which is marketed as *Aspergillus* meal (AM). AM includes 16% protein and 44% fiber and can be used to boost performance in commercial poultry diets with low protein levels [114,115]. The mycelium or *A. oryzae* also contains beta-glucans, FOS, chitosan, and MOS [116,117]. This substance also benefits chickens by promoting growth, most likely by enhancing feed ingredient absorption and digestibility [116].

Beta-glucan is a potent immunity booster [118]. This unique substance affects the intestinal villi and helps the body fight viral and bacterial invaders [119]. MOS bind toxin active sites and defend the GIT against invasion. FOS and chitosan are non-digestible carbohydrates that are easily fermented by gut flora [120]. It was demonstrated that dietary AM alters intestinal morphometry in turkey poults. It increased the number of acid mucin cells, neutral mucin cells, and sulphomucin cells in the duodenum and ileum, in addition to the villi height and surface area in the duodenum and ileum of neonate poults when compared to the control [121]. Another study found that feeding new-born poults AM prebiotic for 30 days boosted body weight and enhanced feed conversion compared to feeding them a basal control diet [122].

Interestingly, dietary AM prebiotic-fed chicks had lower ileum energy and protein content than control chicks, indicating greater digestion and absorption of those nutrients [123]. Fructooligosaccharides (FOS) have been demonstrated to improve intestinal calcium and magnesium absorption, in addition to bone mineral concentrations [124]. Several studies have shown that probiotics can reduce *Salmonella* colonization in hens [110,125,126]. Finally, chitosan is a natural biopolymer created by deacetylating chitin, the major component of fungal cell walls and arthropod exoskeletons. As previously stated, chitosan has several benefits, including antimicrobial and antioxidant properties [127]. In agriculture, horticulture, environmental science, industry, microbiology, and medicine, chitosan has also showed promising applications [117]. Moreover, many studies have used chitosan as a mucosal adjuvant, increasing IgA levels [128].

In another study, the efficacy of 0.2 percent dietary AM against horizontal *Salmonella* spp. transmission was assessed in turkeys and hens [129]. This study found that feeding turkeys and broiler chickens 0.2 percent AM reduced horizontal *Salmonella enterica* serovar Enteritidis transmission and *Salmonella* enterica serovar Typhimurium transmission by reducing overall colonization levels. The reduction in *Salmonella* colonization may be attributed to a synergistic effect of beta-glucan, MOS, chitosan, and FOS found in *Aspergillus oryzae* mycelium.

Yalçın et al. [130] reported that the yeast cell wall derived from baker’s yeast was an effective prebiotic feed additive in broiler feeding due to the increased growth performance, increased humoral immune response, and the reduction in abdominal fat. In another study conducted with laying hens, Yalçın et al. [113] concluded that the yeast cell wall had beneficial effects in the production of low cholesterol eggs and improvement in humoral immunity response.

## 7. Synbiotics

When used in combination with prebiotics, probiotics are termed synbiotics, and have the ability to further improve the viability of the probiotics. Probiotics, prebiotics, and synbiotics are now widely used globally. In the following section, we discuss the role of synbiotics on digestive physiology and poultry production.

### 7.1. Role of Synbiotics in Poultry Production

**Figure 4:**
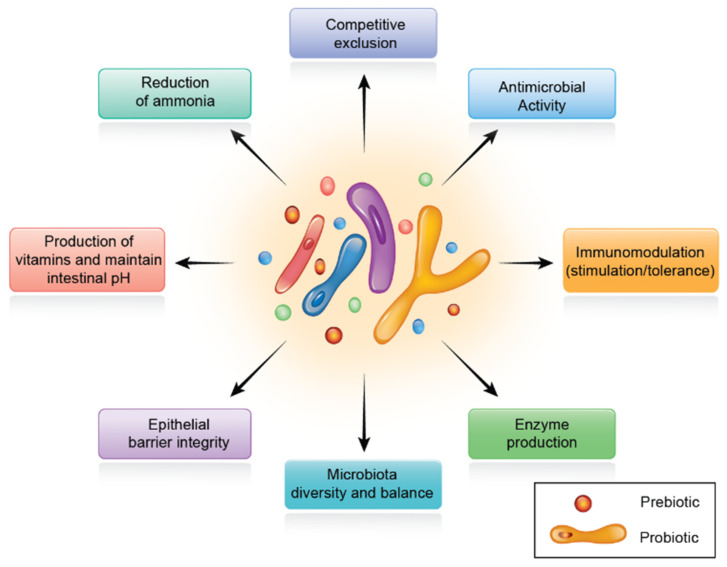
The role of synbiotics on digestive physiology.

Immediately after hatching, birds must switch from endogenous yolk energy to an exogenous carbohydrate-rich diet [131]. During this vital period, intestine size and morphology change dramatically. Changes in epithelial cell membranes alter the mechanical interface between the host’s internal environment and the luminal contents. Studies on early growth nutrition and metabolism in chicks may help optimize nutritional management for optimum growth. The end products digested by symbiotic gut microorganisms can modify not only gut dynamics, but also various physiologic systems [132]. The multiple roles of synbiotics on digestive physiology are summarized in Figure 4.

### 7.2. The Role of Short Chain Fatty Acids (SCFAs) on Digestive Physiology

The principal fermentative response in humans and chickens is the hydrolysis of non-digestible polysaccharides, oligosaccharides, and disaccharides to simple sugars, which are then further fermented by gut bacteria, for example, into SCFAs. In the large intestine, carbohydrate presence and fermentation can change gut physiology. As the intestinal microbiota are established, the SCFA concentration rises from undetectable levels in the ceca of day-old chicks to the greatest concentration at day 15 [133]. The effects of SCFAs are separated into those in the lumen and those in the big gut wall cells. SCFAs are major luminal anions. Increasing their quantities by fermentation reduces the digesta pH to a value of approximately 4.8. SCFAs also provide up to 50% of the daily energy requirements of colonocytes [134]. Fermentable carbohydrates can drastically alter the microbial ecology by providing SCFAs or substrates. However, SCFAs have multiple roles in host and microbial physiology [135].

Recently, the complex metabolic interaction network of a synthetic gut bacterial community (Oligo-Mouse-Microbiota, OMM^12^) was analyzed in detail by in vitro and in vivo methodologies [136]. The study supported the central role of SCFAs in the metabolism of gut bacteria. Hierarchical clustering showed that closely related bacteria in OMM^12^ produced and consumed similar SCFAs. For example, both Bacteroidales strains in OMM^12^ produced acetic acid, succinic acid, and branched-chain SCFAs, whereas butyric acid, valeric acid, and caproic acid were formed by *Clostridium innocuum.* This example underscores the diverse metabolic capabilities of gut bacteria and also reflects the delicate metabolic interplay, balanced substrate usages and competition in microbiota.

#### 7.2.1. SCFAs and Muscular Activity

In vitro, SCFAs at three millimolar dilates precontracted colonic resistance arterioles in different human colonic segments [79]. Acetate, propionate, or butyrate infusions increased intestinal blood flow [137]. SCFAs affect blood flow without prostaglandins or adrenoreceptors. Local neuronal networks, chemoreceptors, and direct impacts on smooth muscle cells are possible modes of action [20]. Colon SCFAs that enter portal circulation appear to alter upper gut musculature. Not only the colon, but the entire gastrointestinal system, depends on these processes. Greater blood flow should improve tissue oxygenation and nutrition delivery [138].

#### 7.2.2. SCFAs and Enterocyte Proliferation

With or without peritoneal delivery, SCFA increases the development of colorectal and ileal mucosal cells in rats. The primary SCFAs (particularly butyrate) appear to reduce the risk of colon cancer [139]. The incorporation of [^3^H]thymidine increased in rats fed deoxycholate plus cholesterol [140]. A low intra-colorectal pH may be the cause of some SCFA effects. Colonocytes cannot take up protonated and insoluble bile acids at pH 6. Inhibition of bacterial conversion of primary to secondary bile acids reduces their carcinogenic potential [141]. Similar results have been shown in broiler chickens where combined intra-amniotic and dietary synbiotic treatments improved broiler intestinal integrity and cecal SCFA production, and increased cecal beneficial bacteria populations [142].

#### 7.2.3. SCFAs and Mucin Production

Endogenous SCFA synthesis by gut bacteria appears to boost mucus formation and release locally. Moreover, the effects of beneficial or probiotic microbes on mucin synthesis have been studied [137]. The capacity of organisms to limit adherence of attaching and effacing organisms to intestinal epithelial cells appears to be mediated by their ability to promote expression of MUC2 and MUC3 intestinal mucins [143]. Probiotics may have broader application than enteropathogen treatments in poultry. Several studies have demonstrated that probiotics increased mucin synthesis, which decreased rotavirus replication, symptoms, and shedding. The proximal colon’s butyrate concentration changed crypt depth and the number of mucus-producing cells. Increased butyrate formation was associated with the number of neutral-mucin-producing cells [125,144].

## 8. Phytogenic Feed Additives

Phytogenic feed additives (PFAs) are classified as sensory and flavoring compounds according to the European Union Legislation (EC 1831/2003) [145]. It has been suggested that PFAs increase the growth performance [146,147], nutrient digestibility [148], and gut health [146,149,150] in poultry. Currently, PFAs are used in feeding programs of poultry and swine. The count of *Lactobacillus* spp. in the caecum was increased when 75 mg/kg red ginseng root powder was added as a feed supplement [151]. Several commercial products are based on herbs such as Anise seeds (*Pimpinella anisum*), Caraway seeds (*Carum carvi*), Cinnamon bark (*Cinnamomum verum*), Chamomile flowers (*Matricaria recutita*), Citrus peel (*Citrus sp*), Clove buds (*Syzygium aromaticum*), Fennel seeds (*Foeniculum vulgare*), Garlic bulbs (*Allium sativum*), Ginger rhizome (*Zingiber officinale*), Melissa leaves (*Melissa officinalis*), Onion bulbs (*Allium cepa*), Oregano leaves (*Origanum vulgare*), Peppermint leaves (*Mentha piperita*), Rosemary leaves (*Rosmarinus officinalis*), Sage leaves (*Salvia officinalis*), Thyme leaves (*Thymus vulgaris*), and Valerian root/rhizome (*Valeriana officinalis*) [152].

These phytogenic substances are promoted due to their safety profiles and qualities to improve the animal performance and health through the following effects: (i) improvement of digestibility, (ii) antimicrobial activities, (iii) anti-inflammatory and antioxidant effects, (iv) stabilization of intestinal microbiota, (v) improvement of animal traits, and (vi) reduction in environmental emissions. In addition to the pharmacological effects, recent studies indicated that phytogenic substances modulate the gut microbiota, namely increase *Firmicutes* [153,154], *Clostridiales*, *Ruminococcaceae*, and *Lachnospiraceae* [155].

Several factors can modulate the intestinal microbiota causing either a positive or negative effect on the host [156]. Dietary effects on the composition of microbiome are shown in Table 2. Supplementation of day-old chickens with antibiotics negatively modulated the intestinal microbiota and adversely affected the immune system development [157]. It was also found that switching the diet from corn-based to wheat- and barley-based led to an increase in *Lactobacillus* and coliform [158]. Water-soluble non-starch polysaccharides increased the viscosity of digestive content and the production of SCFAs, which beneficially regulated ileal motility [159].

**Figure 5:**
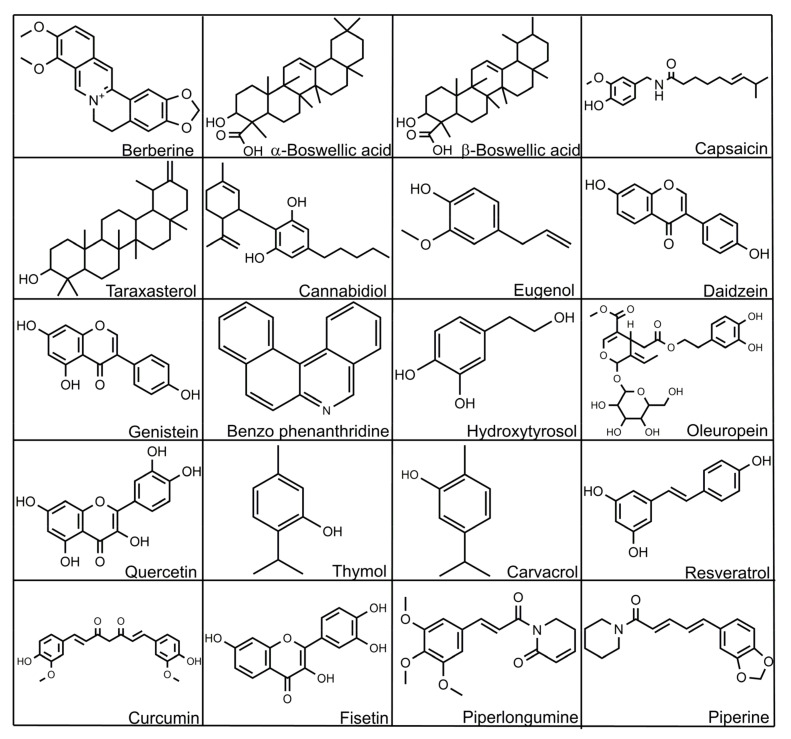
Selected structures of bioactive substances.

In the following section, we discuss the effects of some phytogenic substances that can be used in poultry. Structures of some of these bioactive substances are given in Figure 5.

### 8.1. Berberine

Berberine is an isoquinoline alkaloid found in several medicinal plants, such as *Coptis chinensis Franch*, *Cortex phellodendri,* and *Berberis asiatica* [161]. Like curcumin, berberine has a poor oral bio-availability (less than 1%) [162,163]; however, it has a biological effect on gut microbiota. Several pharmacological effects of berberine have been described, including anti-inflammatory [164,165,166,167], anti-diabetic [164], anti-atherosclerotic [168,169], and cardio-protective actions [170]. Berberine modulates the pro-inflammatory mediators by reduction in TNF-α, IL-1β, and IL-6 [171,172,173,174]. It was found that berberine significantly reduces IL-17A, IL-17F, IL-6, and IL-1β expression and significantly increases IFN-γ and IL-10 in spleens and livers in ducks infected with *Riemerella anatipestifer* [175].

In a mice model, berberine modulates the gut microbiota by increasing *Bacteroides* sp., *Blautia* sp., *Akkermansia* sp., *Lactobacillus* sp., and *Bifidobacterium* sp. [176] and suppression of pathogenic bacteria such as *Escherichia coli* and *Enterococci* sp. [176,177]. In poultry, there may be some correlation between berberine’s effects on growth performance and modulation of gut microbiota composition and functions. Berberine reduced the abundances of *Firmicutes*, *Lachnospiraceae*, *Lachnoclostridium*, *Clostridiales*, and *Intestinimonas* in the gut of broilers, but increased the abundances of the phylum *Bacteroidetes* and the genus *Bacteroides* [178]. Berberine can be also used to control coccidial infection and necrotic enteritis in broilers [179,180]. Berberine was found to be safe for broiler chickens, even at high doses of 1 g/kg in feed [179].

Additionally, the alkaloid was shown to increase the levels of SCFAs, especially under pathological metabolic disorders [176]. In broilers challenged with LPS or *E. coli*, berberine showed antioxidant and anti-inflammatory effects [181,182]. Berberine decreased the mRNA expression of NF-κB, TNF-α, IL-1β, inducible nitrite synthase, and cyclooxygenase-2 in the liver [181]. Interestingly, it was suggested that berberine increases the bioavailability of other drugs in broilers by down-regulation of P-glycoprotein (P-gp) efflux [183].

### 8.2. Boswellia

Trees from the genus Boswellia (*Burseraceae*) are traditionally used as a medicine, a fumigant, in various cosmetic formulations, and in aromatherapy in several countries around the world. Frankincense (*olibanum*) is the common name given to the aromatic resin produced by a group of trees belonging to the genus Boswellia. *Boswellia carteri* Birdw., *B. frereana* Birdw. (Somalia) and *B. serrata* Roxb. (North-western India) are the three main frankincense-producing species [184].

*Boswellia serrata* oleo-gum resin is a traditional Ayurvedic remedy for inflammatory diseases, and is also known as *Salai Guggal, Indian olibanu*, or Indian frankincense. It has a woody, spicy, and haunting smell. The phyto-chemical content of *B. serrata* oleo-gum resin (BSE) is dependent on the botanical origin and consists of 30–60% triterpenes (such as α- and β-boswellic acids, lupeolic acid), 5–10% essential oils, and polysaccharides. The anti-inflammatory properties of *B. serrata* are attributed to the bio-active components, 11-keto-β-boswellic acid (KBA), and 3-acetyl-11-keto-β-boswellic acid (AKBA), even if other boswellic acids, such as β-boswellic acid (βBA), may be efficacious.

It has several mechanisms of action, such as inhibition of 5-lipoxygenase (5-LO), decreased cytokine (interleukins and TNF-α) levels, and reduction in ROS formation [185,186,187]. β-Boswellic acid has an anti-inflammatory activity acting through the inhibition of serine protease cathepsin G and microsomal prostaglandin E synthase [188]. Using Caco-2 cell monolayers, Catanzaro et al. tested the impacts of *B. serrata* oleo-gum extract (BSE) and its pure derivative AKBA at 0.1–10 µg/mL and 0.027 µg/mL, respectively [189]. BSE and AKBA pretreatment significantly prevented functional and morphological alterations in paracellular permeability and the NF-κB phosphorylation induced by inflammatory stimuli. At the same concentrations, BSE and AKBA counteracted the increase of ROS caused by H_2_O_2_ exposure. Together, a positive correlation of the antioxidant activity with the mechanisms involved in the physiologic maintenance of the integrity and function of the intestinal epithelium was demonstrated [189]. They also reported that BSE protects the intestinal epithelial barrier from inflammatory damage in human patients suffering from inflammatory bowel disease (IBD) [189]. In rabbits, *B. serrata* significantly reduced total bacteria counts of *E. coli* and *Salmonella* [190].

In broilers, dietary supplementation of *Boswellia serrata* improved the animal performance by increasing the total antioxidant capacity and the levels of globulin, superoxide dismutase, and digestive enzymes (amylase and lipase), and by reducing the levels of total cholesterol, low-density lipoprotein (LDL), and malondialdehyde (MDA) [191]. It was found that 3% and 4% additions of Boswellia in the diet are safe for broiler chickens and improved the body weight, energy digestibility, and carcass quality [192].

### 8.3. Capsaicin

Pepper (*Capsicum* spp.) is an important vegetable species and a good source of different phytochemicals including vitamin C, phenolic compounds, flavonoids, and carotenoids. Therefore, it has significant antioxidant activities [193,194]. Chili peppers are increasingly used in food and very popular worldwide. Capsaicin is the main bio-active component in red chili (genus *Capsicum*) that provides a pungent flavor to food. Capsaicin has been related to several biological effects, including decreased body fat, anti-inflammatory, anti-carcinogenic, antioxidant activities, and modulation of intestinal motility. 

These actions are mostly due to its role as an agonist of the transient receptor potential vanilloid 1 (TRPV1), expressed in the mesenteric nervous system and epithelial cells of the colon. The anti-inflammatory action of capsaicin is also related to its role in activating the peroxisomal proliferator-activated receptor gamma (PPARγ). Experimental studies suggested that capsaicin could reduce intestinal inflammation by a mechanism that could involve not only the TRPV1 receptor but also PPARγ [195]. Kang et al. reported that dietary capsaicin prevented high fat diet-induced metabolic endotoxemia and systemic chronic low-grade inflammation by elevating cecal butyrogenic bacteria and thus the butyrate levels, inhibiting colonic cannabinoid receptor type 1 (CB_1_) and reducing LPS biosynthesis of bacteria. Therefore, capsaicin prevents gut dysbiosis and metabolic endotoxemia that are linked to chronic inflammatory diseases.

Capsaicin increased the Firmicutes/Bacteroidetes ratio and *Faecalibacterium* abundance that coincided with the increase in the plasma levels of glucagon-like peptide 1 (GLP-1) and gastric inhibitory polypeptide (GIP), and with the decrease in the plasma ghrelin level in healthy Chinese adults [196]. Poultry do not sense the effect of capsaicin, due to the lack of receptors specific for capsaicin binding [197,198] or the lack of receptors that are sensitive to capsaicin [199]. Nevertheless, in broilers, supplementation of 80 mg/kg of natural capsaicin extract in diets was found to be safe and improved animal performance by improving nutrient digestibility, antioxidant status, immune function, and meat quality. Capsaicin extract reduced the concentrations of serum TNF-α and IL-1 β, and increased the total antioxidant capacity of catalase, glutathione peroxidase, and superoxide dismutase [191].

### 8.4. Triterpenoids of Marigold

*Calendula officinalis* L. (marigold) flower extracts were investigated for their antibacterial, anti-inflammatory [200,201,202,203], antitumor-promoting [201], and cicatrizing effects [201], in vitro anti-HIV activity [204], hypoglycemic effects, gastric emptying inhibitory activity, and gastro-protective effects [202]. *C. officinalis* triterpenoids are considered to be the most important anti-inflammatory principles of the extract [200]. Among these triterpenoids, taraxasterol-3-O-myristate (1) and arnidiol-3-O-myristate (2) were shown to modulate stress damages induced by H_2_O_2_ and INFγ + TNFα, underlining the potential use of Calendula extracts against intestinal inflammations [205]. However, in broilers, the supplementation with 5 and 10 g dried powder of *Calendula officinalis*/kg of diet had no positive influence on growth performance [206]. To the best of our knowledge, marigold effects in poultry microbiota have not been studied. Nonetheless, Rajput et al. reported that dietary supplementation with marigold flower extract increases antibody titers against Newcastle Disease virus (NDV) and Avian Influenza virus (AIV), and growth performance of broilers [207]. Thus, more experiments are needed to investigate the probable effects of marigold in broilers on gut health and microbiota population.

### 8.5. Phytocannabinoids

Phytocannabinoids are terpenophenolic C_21_ or C_22_ compounds, which are formed in glandular trichomes of female *Cannabis* flowers [208]. They have important properties, such as regulating food intake, nausea, emesis, gastric secretion, gastroprotection, gastrointestinal tract motility, ion transport, visceral sensation, intestinal inflammation, and cell proliferation in the gut [209,210]. Recently, their potential modulatory activity in GIT has attracted considerable attention. Konieczka et al. [211] reported that cannabidiol (CBD) and nano-selenium improved the gut barrier functions in chickens through increased expression of genes controlling gut integrity. Moreover, CBD and nano-selenium may be able to modulate the response of chickens to *C. perfringens* infection, which may allow time for effective intervention [208]. Using the Caco-2 cell culture model of intestinal permeability, it was concluded that the cannabinoids may play a role in modulating intestinal permeability by increasing the TJ protein zona occludens-1 [212].

### 8.6. Eugenol

Eugenol is a volatile phenolic constituent of clove essential oil obtained from *Eugenia caryophyllata* buds and leaves, mainly harvested in Indonesia, India, and Madagascar. 1,2-Eugenol is the main constituent (70–90%) of clove oil and is responsible for the clove aroma [213]. Clove oil has antimicrobial, antifungal, antiviral, antioxidant, anti-inflammatory, and anticancer properties [214]. Authors found that clove essential oil significantly modulated global gene expression and altered signaling pathways critical for inflammation, tissue remodeling, and cancer signaling processes. The results of this study also suggest clove essential oil is an anti-inflammatory, immune-modulating, and tissue remodeling agent in poultry. A recent study reported positive effects of a microencapsulated product composed of eugenol on performance under a subclinical necrotic enteritis [215]. However, the mode of action of plant extracts in mitigating necrotic enteritis effects on intestinal health is not well documented. In another recent study, it was also noted that birds fed eugenol and garlic had reduced CLDN5 expression in male birds, and *Bacteroides* spp. in female birds, than in the control group [216], suggesting that eugenol and garlic supplementation mitigates the effect of necrotic enteritis by improving the intestinal health of birds.

### 8.7. Isoflavones (ISF)

During the last decade, scientists have paid more attention to isoflavones (ISFs), for example, daidzein, genistein, and glycitein, due to their noticeable benefits to human health [217]. Indeed, several studies showed that ISFs have antioxidant properties [218], can enhance the immune system [219], prevent breast cancer, lower the risk of osteoporosis, decrease the plasma cholesterol level, and boost the anti-oxidative potential in humans and animals [220]. Bacteria that colonize the digestive tract are known to modify ISFs, which are represented in plants as both glycosides and aglycones. Before ISFs can be absorbed from the gut, the sugars of the glycosides must be deconjugated by β-glucosidases expressed by intestinal bacteria and, subsequently, ISFs enter the bloodstream via passive absorption [221]. Mammalian β-glucosidase activity does not appear to substantially contribute to deconjugation of ISF glycosides in monogastric animals due to its lower expression level [222]. Supplemental 10–20 mg/kg ISF may have a positive effect on broiler chickens infected with infectious bursal disease virus, probably because ISFs decrease the severity of bursa lesions and viral protein 5 mRNA expression, a protein produced in response to IBDV to drive apoptosis, and have strong antioxidant activity [223]. One study highlighted the positive benefits of an ISF-rich diet on broiler chickens suffering from the infectious bursal disease virus. Dietary ISF improved the overall health and condition of infected chickens. Further antioxidative properties of ISFs in male broilers are described by the consumption of 40 or 80 mg ISF per kg bodyweight, which leads to an increased antioxidant capability and superoxide dismutase activity in plasma [218].

As a dietary supplement, ISF has recently gained popularity, especially for late-laying stages of egg production that require hormonal replacement to increase production. It has been suggested that the level of endogenous estrogen, individual variation, duration, and dose of phytoestrogen are factors affecting its effects on estrogen [224]. According to several studies, ISF improved animal growth and reproduction [218,225,226,227]. Furthermore, Shi et al. [227] reported that feeding ISF to laying hens at 59 weeks of age resulted in an increase in egg production. Additionally, dietary daidzein at 10, 20, and 30 mg/kg increased egg weight and fertility [228]. Moreover, a diet containing daidzein significantly improved the productivity of Shaoxing duck breeders during late laying [229]. Improvements in eggshell quality and laying performance were also observed in the post-peak laying stage of hens [230]. Furthermore, feeding quails during the late laying stage significantly improved egg quality and bone mineralization [231].

Calcium ions are essential components in bone and eggshell formation. Studies have shown that ISF decreases Ca^2+^ concentration in osteoclasts [232], and as a result, more Ca^2+^ is available for the eggshell formation process. Studies conducted by Zhao et al. [229] and Sahin et al. [226] confirmed that chicken and quail eggshells develop better when the diet was supplemented with the ISF daidzein.

### 8.8. Isoquinoline Alkaloids

Herbal extracts from *Macleaya cordata* (plume poppy) are rich in isoquinoline alkaloids (IQAs), particularly in benzo phenanthridine and protopine alkaloids such as sanguinarine [233]. Le et al. [234] reported that IQAs prevented in heat-stressed pigs the increases in macromolecule permeability of the intestine, and therefore protected mucosal integrity. Beneficial effects of sanguinarine on reducing colonic leakiness have also been demonstrated in a model of colitis in rats [235]. Robbins et al. [236] found that integrity was improved in pigs fed a diet containing 1.5 mg of benzo[c]phenanthridine alkaloids per kg of feed. The potential mechanisms of action of QBAs are not currently known, but may include modulation of gastrointestinal microbiota, enhancement of intestinal protection, and repair of the intestinal epithelium. Liu et al. [237] reported that the introduction of *M. cordata* extract supplements to the pig’s diet increased volumes of ZO-1 and claudin-1. The findings of this study indicated that *M. cordata* extract enhances intestinal barrier function in growing piglets and that it can be used as a viable substitute for antibiotics. To the best of our knowledge, the IQA effects in poultry have not been studied. Nonetheless, IQA supplementation may be used as a nutritional strategy to improve gut health and prevent the occurrence of a leaky gut, thereby maximizing the usage of nutrients for performance of pigs, but probably also of poultry.

### 8.9. Phenolic Derivatives

Olive tree (*Olea europaea* L.) is one of the most relevant botanical drugs in traditional Mediterranean medicine, and olive leaf extracts have been used with different purposes including anti-hypertensive, anti-atherogenic, anti-inflammatory, hypoglycemic and hypocholesterolemia activities [238]. These extracts contain many potentially bio-active compounds, especially phenolic derivatives, such as phenolic acids, phenolic alcohols (hydroxytyrosol), flavonoids, and secoiridoids (oleuropein) [239]. Olive oil phenolic compounds contribute to maintaining gut barrier integrity by upregulating the expression of genes involved in maintaining tight junctions between intestinal cells, modulating the oxidative status of the intestinal epithelial layer, in addition to the inflammatory and immune response [240,241,242]. In addition, phenolic compounds extracted from olive leaves may be beneficial to broilers through their antimicrobial activity against intestinal pathogenic bacteria [243]. *Firmicutes* was the predominant phylum in the caeca of broiler fed diets contained 750 ppm of an olive pomace [244]. Liu et al. showed that administration of hydroxytyrosol to high fat diet-induced obese mice increased gene expression for the tight junction-associated proteins ZO-1 and occludin [245], and reduced levels of plasma lipopolysaccharides and inflammatory cytokines in the liver [245]. Thereby, it was concluded that hydroxytyrosol has an important role in promoting intestinal barrier integrity. Vezza et al. [246] reported that olive leaf extract supplementation improved the epithelial barrier function in the models of experimental colitis as demonstrated by the increased expression of the mucin MUC-2, the tight junction protein ZO-1, and TFF-3 [246]. They concluded that intestinal anti-inflammatory activity of olive leaf extract in colitis mouse models may be related to its immunomodulatory properties and the capacity to restore the intestinal epithelial barrier.

### 8.10. Quercetin

Quercetin is the most common flavonoid in nature and can be found in fruits and vegetables including onions, kale, and apples. Quercetin in onion peel has higher bioavailability than that of apple peel [247]. It is one of the most investigated polyphenols exhibiting various health-promoting properties, for example, antimicrobial, antioxidative, anti-inflammatory, and metabolic effects [248]. Quercetin induces their antibacterial activity by acting as DNA gyrase on various cell targets, bacterial membrane and motility, type II fatty acid biosynthesis pathway, and D-alanine:D-alanine ligase enzyme inhibitor [249,250]. Abdel-Latif et al. observed that the total coliforms and *C. perfringens* were decreased (*p* < 0.05) in quercetin-supplemented groups (200–800 ppm). Conversely, *Lactobacillus* counts were increased (*p* < 0.05), due to improvement in the gut microbiota environment in quercetin-supplemented groups [251]. Quercetin is a flavonoid that has been proposed to exert beneficial effects over the intestinal barrier function in human intestinal Caco-2 cell monolayers. Suzuki and Hara [252] reported that quercetin enhances the intestinal barrier function through the assembly of zonula occludens (ZO)-2, occludin, and claudin-1 by inhibiting protein kinase Cδ (PKCδ). The increase in claudin-4 expression has an additional role after 12 h. Amasheh et al. [253] evaluated the effects of quercetin on cytokine-induced intestinal barrier damage, both in HT-29 cells and in the distal colon from male Wistar rats ex vivo. Quercetin exerts a protective effect on the intestinal barrier by down-regulating claudin-2. The analysis of intestinal permeability in rat colon ex vivo revealed that quercetin partially inhibited the effects of TNF-α and IFN-γ that reduced the total resistance of the intestinal barrier [253]. Carrasco-Pozo et al. [254] evaluated the protective effect of quercetin on ZO-1 and occludin in Caco-2 cells treated with indomethacin and rotenone (an environmental toxin). Treatment with quercetin protected ZO-1 delocalization and prevented the decrease in ZO-1 and occludin expression. The authors hypothesized that quercetin’s effects may be due to its mitochondrial-protecting property. However, it may also be the result of a modulatory effect of quercetin on the activity of various intracellular signaling molecules that regulate the integrity of TJ. Quercetin inhibited isoform-mixed protein kinase C (PKC) [255] and phosphoinositide-3-kinase (P13K) [256].

### 8.11. Thymol/Carvacrol

**Figure 6:**
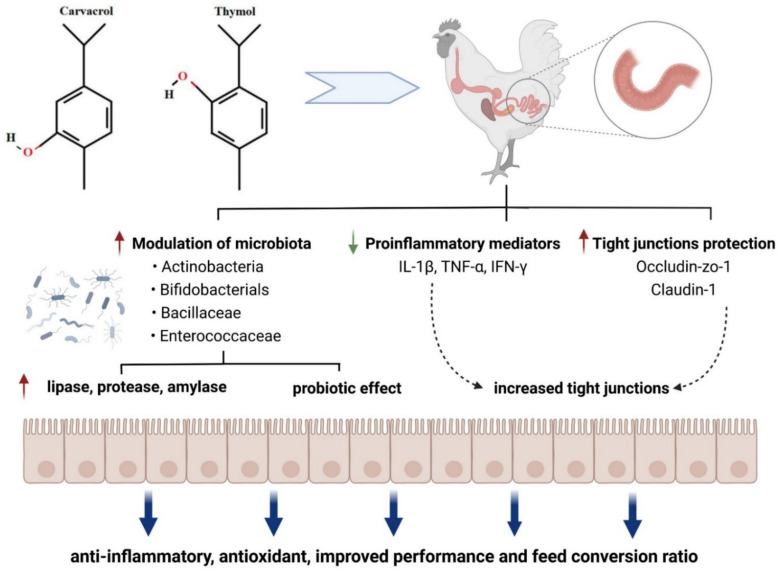
Modulation of gut microbiota by carvacrol and thymol and their biological effects, modified from Feng et al. [270] (figure was created with BioRender.com, accessed on 15 December 2021).

Oregano and thyme are members of the *Lamiaceae* family, an aromatic herb used extensively in food to add a distinctive aroma and flavor. Their active principles are reported to have antihelminthic, antiseptic, expectorant, antispasmodic, antifungal, antimicrobial, immunostimulating, hypocholesterolemia, antioxidative, antiviral, carminative, sedative, and diaphoretic effects [257,258,259]. *Thymus vulgaris L.* contains 1–2.5% essential oil containing monoterpenes, mainly thymol and its phenol isomer carvacrol. Phenolics in essential oil, such as caffeic acid and p-cymene-2,3-diol, and some biphenylic and flavonoid compounds, such as flavonoid glycosides and flavonoid aglycones, are assumed to contribute various beneficial effects in animals [257,258,260]. Modulation of gut microbiota by carvacrol and thymol and their biological effects are shown in Figure 6.

In the study of Turner [261], thyme oil supplementation to rabbit diets increased TEER values of the intestinal wall. This result showed that thyme oil supplementation has a positive effect on the intestinal barrier. Placha et al. [262] also reported that thyme oil may strengthen the intestinal barrier. Dietary supplementation with 0.5 g/kg dry matter thyme oil may improve intestinal integrity. Yalçın et al. [260] showed antioxidative and hypolipidemic effects of thyme supplementation in laying hens along with improved humoral immune response without negative effects on performance and egg quality characteristics. Yoshino et al. [263] reported that oregano extract exhibited iron-reducing activity, although its strength was approximately one-fifth of that of ascorbic acid. Oregano extract administration prevented mouse gastritis induced by cold-restraint stress. The antioxidant activities of oregano extract appear to contribute to its preventive effects against inflammatory diseases such as stress-induced gastritis in mice. Han and Parker [264] reported that oregano essential oil inhibited the levels of many inflammatory and tissue remodeling biomarkers, including MCP-1, VCAM-1, ICAM-1, IP-10, ITAC, IP-10, MIG, collagen I, collagen III, M-CSF, EGRF, MMP-1, PAI-1, TIMP1, and TIMP2. With the analysis of genome-wide gene expression, it was also shown that oregano essential oil exerted a robust and diverse impact on many genes and signaling pathways, many of which are critically involved in inflammation, tissue remodeling, and cancer signaling processes. Oregano essential oil, having carvacrol as the major active component, is a promising candidate for use in skin care products with anti-inflammatory and anticancer properties [264]. Avola et al. reported that oregano essential oil has a property of treating inflammation and supporting cell motility during wound healing in a human keratinocytes cell model [265]. Both thymol or carvacrol up-regulated the mRNA expression of occludin, ZO-1, and claudin-1 in the small intestine of broiler chickens [266,267], strengthening the tight junctions. Additionally, thymol and carvacrol improved the digestive enzyme activities through increasing the activities of amylase, protease, and lipase [268]. The increased digestive enzyme activities may be attributed to their antibacterial activity and modulatory effects on gut microbiota [269], resulting in less pathogen-induced damage of enterocytes.

### 8.12. Resveratrol

Resveratrol is a naturally occurring polyphenolic compound found in various plants, including grapes, *Polygonum cuspidatum*, and *peanuts* [271]. It has several biological effects, including anti-inflammation [272,273], anti-oxidation [272,274], and energy metabolism regulation [275]. Mayangsari and Suzuki [276], and Blaster [16], found that resveratrol can protect the integrity of human Caco-2 colonic epithelial cells’ tight junctions and improve intestinal epithelial barrier function. Zhao et al. [277] reported that resveratrol can maintain the intestinal barrier’s integrity and reduce intestinal damage by inhibiting the apoptosis of intestinal epithelial cells of rats. In broilers reared under normal ambient temperature, resveratrol can improve the muscle antioxidant function [278]. Moreover, resveratrol exerted beneficial effects on intestinal morphology [271], the spleen, and muscle antioxidant capacity [271] of broilers under heat stress. The mechanism by which resveratrol enhances the intestinal antioxidant capacity is mediated by the Nrf2 signaling pathway [279]. Resveratrol modulated the gut microbiota by increasing the *Lactobacillus* sp., *Bifidobacterium* sp. Bacteroidetes, *Akkermansia* sp. and *Ruminococcus* sp., whereas the levels of *Lactococcus* sp., *Clostridium* spp., *Oscillibacter* spp., *Hydrogenoanaerobacterium* sp. were reduced [280]. It improves the tight junction and reduces the permeability of LPS [281,282].

### 8.13. Curcumin

Curcumin is a major active component of the food flavor turmeric, isolated from the powdered dry rhizome of *Curcuma longa* Linn. Curcumin is characterized by the following: (i) based on FDA, WHO, and EFSA, oral administration is safe and the ADI is 0–3 mg/kg [283]; (ii) it is highly resistant to low pH, not metabolized in the stomach [284]; (iii) it is absorbed from the large intestine and detected in blood as glucuronide conjugates and sulfate conjugates; (iv) it is metabolized in enterocytes and hepatocytes by reductase to di-, tetra-, and hexa-hydrocurcumin [285,286,287]; and (v) it shows poor gastrointestinal absorption and low bioavailability, mainly attributed to water insolubility, and rapid metabolism and excretion [288]. In rats, about 75% of curcumin was excreted in the feces, and a very low amount was detected in the urine [289]. Natural products such as piperine, in addition to nano-formulations, increased curcumin bioavailability [290,291].

Curcumin is said to have a variety of pharmacological activities, including antioxidative, anti-inflammatory, anti-carcinogenic, antidiabetic, and anti-HIV effects [292]. It was shown that curcumin induces several endogenous antioxidants in cultured intestinal disorders and reduces mucosal injury in trinitrobenzene sulfonic acid-induced colitis in vivo [293,294]. Some studies [295,296,297] reported that curcumin has cytoprotective properties by inducing the protective protein Heme oxygenase-1 (HO-1). Curcumin prevented TNF-α-induced decrease in zonula and occluden-1 (ZO-1) protein levels in Caco-2 cell layers [294].

It was proposed that curcumin exerts its main regulative effects primarily in the gut, especially following oral administration of high doses [237]. Interestingly, the interaction between curcumin and gut microbiota is bidirectional. On the one hand, gut microbiota enzymes play a role in the metabolism of curcumin through reduction, acetylation, hydroxylation, demethylation, and demethoxylation [298]. On the other hand, curcumin modulates the gut microbiota, improves intestinal barriers models [46,293,294,299], and counteracts pro-inflammatory mediators [300]. Curcumin reduced *Ruminococcus* species that are linked with colorectal cancer (CAC) in the mice model, and increased the relative abundance of *Lactobacillales* and decreased the fraction of *Coriobacterales* [301,302].

Curcumin can thus be helpful for treatment of intestinal disorders through the following effects: (i) It protects intestinal epithelial cells against H_2_O_2_-induced disruption of tight junction (TJ) and barrier dysfunction via the HO-1 pathway. (ii) It restores occludin enzyme and ZO-1 protein levels after H_2_O_2_ treatment. Its effects were tested on Caco-2 cells and HT-29 cells, and it was found that curcumin can reduce the disruption of intestinal epithelial barrier functions [303]. (iii) Curcumin can also reduce the release of IL-1b secreted by LPS, induce IEC and macrophages, and prevent the disintegration of tight junction proteins, such as ZO-1, claudin-1, claudin-7, and actin filaments [46]. Therefore, it was concluded that curcumin is a potential compound for treating intestinal barrier injury through increasing the expression of tight junction proteins. In specific pathogen-free (SPF) chickens experimentally infected with *Eimeria maxima*, curcumin reduced the enteric isoprostane 8-iso-PGF2α and prostaglandin GF2α. Additionally, it proved to be effective to reduce Salmonella enterica serovar *Typhimurium* intestinal colonization and maintain better intestinal homeostasis in chickens [304]. To improve the bioavailability of curcumin, nanocapsules may be a future strategy.

## 9. Conclusions

It is critical in modern animal production systems to shift the status from survival to creation; that is, minimize the impacts of chronic inflammation and excessive stress so that chickens can utilize their energy for growth rather than defense. Although there is no “magic bullet” for preventing the multifactorial conditions associated with chronic stress, numerous studies have shown that alternative products, such as probiotics, direct-fed microbials, prebiotics, and phytochemicals, can help to improve intestinal microbial balance, metabolism, and gut integrity. These feed additives have been demonstrated to have anti-inflammatory, antioxidant, immunological modulatory, and barrier integrity-enhancing characteristics. As far as we are aware, no harmful effects have been reported with the use of the nutraceuticals in poultry discussed in the present review. To meet their health and productivity goals, poultry farmers who have eliminated antibiotics from their production systems may utilize a combination of alternative products in conjunction with enhanced management methods, rigorous biosecurity, and effective immunization programs. The relevance of dietary items and their quality, in addition to the absence of *Mycoplasma* spp. and *Salmonella* spp. from genetic lines, cannot be overstated. Any kind of stress induces intestinal inflammation, oxidative stress, and lipid peroxidation of vital cellular components, such as the cell and mitochondrial membranes. Damage to these organelles compromises cell homeostasis and the birds’ health and productivity. All animals have efficient mechanisms to avoid oxidative stress, such as glutathione peroxidase or superoxide dismutase. Nevertheless, chronic stress and chronic inflammation can overload the bird’s system. Antibiotic-free poultry production systems employ alternative natural products, such as those discussed in this review, to reduce the effects of inflammation, colonization risk, and transmission of food-borne pathogens. These products also serve as strategies to maintain human and animal health and food safety in poultry production systems.

## Figures and Tables

**Table 1:** Potential biomarkers to evaluate intestinal health.

Measurement/Function	Biomarker Type
Antioxidant activity	Superoxide dismutase (SOD), Thiobarbituric acid reactive substances (TBARS), Total antioxidant capacity
Gene expression of host protein biomarkers and tight junction	Fatty acid binding protein (FABP), Fibronectin, Occludin, Zonula occludens, Claudins
Immune activity	Acute phase proteins, Calprotectin, Lipocalin, Immunoglobulins (IgA), Interferon gamma (INF-γ)
Intestinal permeability	Fluorescein isothiocyanate dextran (FITC-d), Trans epithelial electrical resistance (TEER), Bacterial translocation
Enterocyte function	Extracellular signal-regulated kinase (ERK), Citrulline

Adapted from Chen et al. [54] and Baxter et al. [59].

**Table 2:** Dietary effect on the composition of the microbiome.

Enterotypes	Biological Activities	Favorable Substance/s
*Bacteroides*	saccharolytic, proteolytic biotin, riboflavin, pantothenate and ascorbate synthesis	proteins and fats
*Prevotella*	mucin/glycoprotein degrading thiamine and folate synthesis [2].	high fiber diet
*Ruminococcus*	mucin/glycoprotein degrading. transmembrane transport of sugars	Sugars

Adapted after [22,160].

## Data Availability

Not applicable.
